# Usage of Silver Nanoparticles in Orthodontic Bonding Reagents

**DOI:** 10.3390/jfb16070244

**Published:** 2025-07-03

**Authors:** Janet Jisoo Lee, Meigan Niu, Zinah Shakir, Geelsu Hwang, Chun-Hsi Chung, Mark S. Wolff, Zhong Zheng, Chenshuang Li

**Affiliations:** 1 School of Dental Medicine, University of Pennsylvania, Philadelphia, PA 19104, USA; janjlee@upenn.edu (J.J.L.); meigan@upenn.edu (M.N.); zinahshakir@gmail.com (Z.S.); 2 Department of Preventive and Restorative Sciences, School of Dental Medicine, University of Pennsylvania, Philadelphia, PA 19104, USA; geelsuh@upenn.edu; 3 Department of Orthodontics, School of Dental Medicine, University of Pennsylvania, Philadelphia, PA 19104, USA; chunc@upenn.edu; 4 Division of Restorative Dentistry, School of Dental Medicine, University of Pennsylvania, Philadelphia, PA 19104, USA; mswolff@upenn.edu; 5 Department of Periodontics, School of Dental Medicine, University of Pennsylvania, Philadelphia, PA 19104, USA

**Keywords:** orthodontics, composite, glass ionomer, sliver, nanoparticle

## Abstract

Fixed orthodontic appliances, which are cemented to tooth surfaces, complicate the maintenance of oral hygiene and create a rough surface that is favorable for bacteria attachment. Additionally, the presence of orthodontic appliances may conceive a unique environment that interacts with cariogenic microorganisms, fostering a distinct microbial ecosystem compared to that of the patients without orthodontic appliances, thus increasing the vulnerability of tooth surfaces to demineralization and caries formation. Silver (Ag) has shown strong antimicrobial effects and has been extensively investigated in the medical field. Here, we aim to review the antibacterial properties and potential side effects of silver nanoparticles (AgNPs) when incorporated into orthodontic bonding reagents. This valuation could contribute to the development of novel bonding reagents designed to prevent the formation of white spot lesions and caries during orthodontic treatments.

## 1. Introduction

Orthodontics is a specialized branch of dentistry that focuses on correcting occlusal alignment and enhancing facial aesthetics, which can significantly improve patients’ quality of life in collaboration with other specialists [1]. To date, fixed orthodontic appliances, such as braces, remain the most commonly used option, especially for more complex cases, and are generally considered more effective than removable alternatives. However, because fixed orthodontic appliances are cemented on the tooth surface, they can complicate oral hygiene maintenance [2] and create a rough surface that promotes bacterial attachment [3]. As a result, fixed orthodontic appliances may not only trap food remnants and microbial secretions, such as toxins, tissue-damaging enzymes, and acidic byproducts, but also promote dental plaque accumulation. Consequently, it can create a favorable environment for pathogens to evade the host’s immune defenses and resist medications [4]; the use of fixed orthodontic appliances may contribute to oral-dental problems such as caries and periodontal inflammation [5,6]. Furthermore, regardless of whether they are fixed or removable, orthodontic appliances can foster a unique environment that interacts with cariogenic microorganisms, resulting in a distinctive microbial flora for patients wearing these devices compared to those who do not [7,8]. This altered microbial ecosystem increases the susceptibility of tooth surfaces to demineralization and caries development while also elevating the risk of periodontal damage, such as gingival inflammation and alveolar bone resorption [5,9]. To address these challenges, clinicians and researchers have dedicated significant efforts to developing innovative dental materials that offer antimicrobial benefits while remaining biocompatible [10].

Among the various materials and agents explored, silver has emerged as a leading option due to its strong antimicrobial properties [8,10,11,12,13,14]. Notably, both metallic and ionic forms of silver exhibit antimicrobial potency [8,10]. Silver ions, in particular, target various bacterial structures. For example, silver ions bind to bacterial cell walls and cytoplasmic membranes through electrostatic attraction and their affinity for sulfur proteins, increasing membrane permeability and disrupting its integrity [15,16,17]. As a result, the disruption leads to RNA and DNA degeneration. Additionally, silver ions can also interfere with amino acid function and impair cellular respiration [17]. Due to the complexity of silver’s anti-microbial mechanism, mutations in at least three key systems are necessary for microbes to develop silver resistance [12,13]. Therefore, silver is effective in treating and preventing multiple diseases caused by drug-resistant microorganisms [10]. Furthermore, silver is abundantly available in nature and relatively cost-effective, making it an appealing substance for scientists to harness its potential in dental treatment [8,18].

Meanwhile, the particle size of a given agent significantly influences its antimicrobial effectiveness [19,20,21]. Advances in nanotechnology have enabled the production of various materials at the nanoscale, with nanoparticles (NPs) typically measuring less than 100 nm [22]. Owing to their large surface area-to-volume ratios, NPs can effectively release ions from their constituent materials, which disturbs microorganisms’ cell function. Moreover, NPs can attach and penetrate the cell walls of Gram-positive and Gram-negative bacteria, and have thus been considered a more effective formulation of antibacterial agents [18].

In particular, silver nanoparticles (AgNPs) exhibit exceptionally high antimicrobial activity against a broad range of microorganisms, including bacteria, viruses, and fungi, outperforming many other antimicrobial agents [16]. Thus, AgNPs have been employed as the primary component in inorganic and polymeric-based antimicrobial (nano)composites to prevent biofilm formation, combat infection, and promote caries arrestment [16,23]. AgNPs are considered relatively safe bactericidal agents so far, and they can act synergistically with several antibiotics [24]. More promisingly, AgNPs have been found to induce osteogenesis [12,24,25]. As a result, AgNPs have been broadly incorporated into dental materials, such as dental implants and root canal irrigation [24,26].

To date, numerous attempts have been made to utilize AgNPs in orthodontic appliances [27]. However, a gap still exists between bench-top investigation findings and their clinical application. Furthermore, the impacts of AgNPs on the biomedical, physical, and mechanical properties of orthodontic materials require a thorough assessment. Since incorporating AgNPs in orthodontic bonding reagents has been investigated extensively in the past two decades, here, we aim to consolidate existing knowledge to facilitate further advancements in developing innovative, multipotent orthodontic bonding reagents.

## 2. Materials and Methods

**Figure 1:**
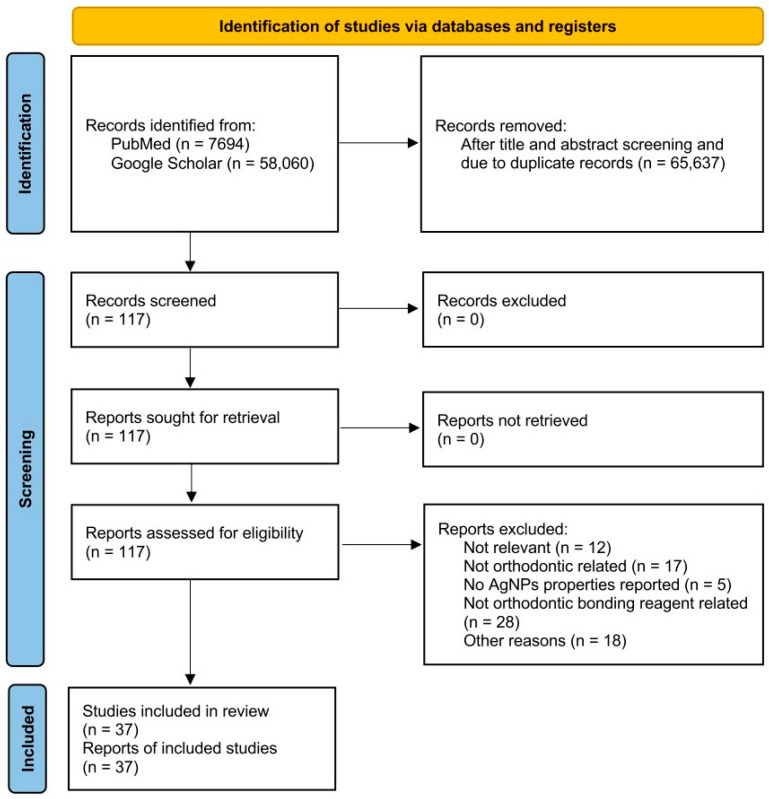
The PRISMA flow diagram of current review.

Original studies reporting the AgNPs in orthodontic bonding reagents were identified through comprehensive searches of PubMed and Google Scholars, using the following keywords: “silver nanoparticles”, and “orthodontics”, “orthodontic materials”, “orthodontic bonding”, “orthodontic composite”, “orthodontic cement”, “orthodontic adhesive”, “glass ionomer”, or “resin-modified glass ionomer cement”. The literature search, completed in March 2025, included only peer-reviewed, English-language original studies published since year 2010, and excluded conference abstracts, editorials, opinions, preprints, thesis, and literature reviews. To avoid missing any related articles, each database was searched independently by two authors. The data extraction from each original article was also performed by two authors separately. The information being extracted from each article includes the type of bonding reagent used, the type and concentrations of AgNPs, the type of study, the reported antibacterial effect, and any reported side effects. In cases of disagreement, a third author was involved to verify the accuracy of data extraction. This study is registered on Open Science Framework (osf.io/8nxp7), and the PRISMA Extension for Scoping Reviews (PRISMA-ScR) checklist was employed as the guideline. The PRISMA flow diagram is shown in Figure 1.

## 3. Nanosilver Particles in Orthodontic Primers

### 3.1. Antibacterial Effects

AgNPs have been incorporated into various bonding primers (Table 1 and Table 2). Notably, even at a low concentration of 0.11% (*w*/*w*), the inclusion of AgNPs in the Transbond^TM^ XT primer (3M Unitek Corp., Monrovia, CA, USA) [28] and Universal bonding (Dentonics Inc., Monroe, LA, USA) [29] significantly inhibited the growth of *Streptococcus mutans*, an indicator cariogenic bacterial species often prevalent during orthodontic treatment [30] (Table 1).

**Table 1:** The antibacterial effects of the silver nanoparticle (AgNPs)-added bonding primers. Conc.: concentration; *w*/*w*: weight per weight; HA: hydroxyapatite; N/A: not applicable.

Bonding Reagent	References	Tested Conc. of AgNPs (*w*/*w*)	Combinatory Materials	Type of Study	Antibacterial Effect
Transbond^TM^ XT primer (3M, Monrovia, CA, USA)	Degrazia et al., 2016 [28]	0.11%, 0.18%, 0.33%	N/A	In Vitro	All Conc. inhibit *S. mutans*
Transbond^TM^ XT primer (3M)	Blocher et al., 2015 [31]	0.11%, 0.18%, 0.33%	N/A	In Vitro	N/A
Universal Bonding (Dentonics Inc., Monroe, LA, USA)	Jenabi et al., 2023 [29]	0.5%, 1%, 2.5%, 5%	N/A	In Vitro	All Conc. inhibit *S. mutans*
Transbond^TM^ XT primer (3M)	Akhavan et al., 2013 [32]	1%, 5%, 10% Ag-HA	Doped HA with various concentration of Ag nanoparticle	In Vitro	N/A
Single Bond™ Universal Adhesive (3M)	Gilani et al., 2020 [33]	1%, 5%, 10% Ag-HA	Ag-HA nanoparticle powder	In Vitro	N/A

### 3.2. Side Effects

#### 3.2.1. Influence on Shear Bond Strength (SBS)

Degrazia et al. reported that lower concentrations of AgNPs, ranging from 0.11 and 0.33% (*w*/*w*), also markedly reduced the SBS of the Transbond^TM^ XT primer, although the resulting SBS values remained above the clinically accepted threshold of 6–8 MPa [28] (Table 2). In contrast, Blocher et al. found that, within the same concentration range, neither silver microparticles (3.5–18 μm) nor AgNPs (12.6–18.5 nm) considerably altered the SBS of the Transbond^TM^ XT primer after the bonded brackets were stored in distilled water at 37 °C for 24 h [31]. Thus, whether the incorporation of AgNPs at these low concentrations genuinely impacts the clinical bonding performance of the Transbond^TM^ XT primer warrants further validation.

Regarding Universal Bonding primer, Jenabi et al. noted that their SBS values decreased with the concentration of incorporated AgNPs [29]. However, this study was conducted using a combination of an AgNP-modified Universal Bonding primer and an AgNP-modified composite [29]. As a result, it remains unclear whether using AgNP-modified Universal Bonding primers with a standard, unmodified composite would yield SBS values that meet clinically acceptable levels.

The effects of modified AgNPs, specifically silver-hydroxyapatite (Ag-HA) nanoparticles, on SBS have also been investigated. Hydroxyapatite (HA), a calcium phosphate compound, prevents calcium loss from demineralized enamel, enhancing tooth resistance to cavities [33]. HA alone can also inhibit the growth of *S. mutans* without disturbing the SBS of orthodontic composites [34]. In previous studies, 1%, 5%, and 10% (*w*/*w*) Ag-HA nanoparticles were mixed with either Transbond^TM^ XT primer [32] or Single Bond™ Universal Adhesive (3M) [33], respectively. Interestingly, the same concentration of Ag-HA nanoparticles exhibited contrasting effects depending on the primer type. For instance, 1% Ag-HA nanoparticles increased the SBS of Transbond^TM^ XT primer but decreased that of Single Bond™ Universal Adhesive, while 10% Ag-HA nanoparticles reduced the SBS of Transbond^TM^ XT primer but enhanced that of Single Bond™ Universal Adhesive. Unfortunately, neither study provided data on the antibacterial effects of these Ag-HA nanoparticle-modified primers. Therefore, the currently available evidence is short for defining the optimized concentration of Ag-HA nanoparticles that can provide promising antibacterial effects while maintaining or even improving the SBS.

#### 3.2.2. Discoloration

One major disadvantage of Ag particles is their dark color, which can compromise esthetics [31], especially in dental applications. For example, Blocher et al. compared the impacts of incorporating silver microparticles and AgNPs into the Transbond^TM^ XT primer. They found that the addition of AgNPs led to more pronounced discoloration after debonding compared to silver microparticles or no-silver controls [31], highlighting the aesthetic concerns that AgNP-modified adhesives may heighten the risk of enamel discoloration, which potentially hinders their suitability for dental applications where appearance is a priority.

**Table 2:** The side effects of the silver nanoparticle (AgNPs)-added bonding primers. Conc.: concentration; *w*/*w*: weight per weight; SBS: shear bonding strength; HA: hydroxyapatite; N/A: not applicable.

Bonding Reagent	References	Tested Conc. of AgNPs (*w*/*w*)	Combinatory Materials	Type of Study	Side Effects		
					SBS	Cytotoxicity	Discoloration
Transbond^TM^ XT primer (3M)	Degrazia et al., 2016 [28]	0.11%, 0.18%, 0.33%	N/A	In Vitro	Decreased with all Conc.	N/A	N/A
Transbond^TM^ XT primer (3M)	Blocher et al., 2015 [31]	0.11%, 0.18%, 0.33%	N/A	In Vitro	No significant difference	N/A	All Conc. showed silver spots under 10× Magnification
Universal Bonding (Dentonics Inc.)	Jenabi et al., 2023 [29]	0.5%, 1%, 2.5%, 5%	N/A	In Vitro	Dose-dependently decreased *	N/A	N/A
Transbond^TM^ XT primer (3M)	Akhavan et al., 2013 [32]	1%, 5%, 10% Ag-HA	Doped HA with various concentration of Ag nanoparticle	In Vitro	1%: significantly increased; 5%: no significant difference; 10%: significantly reduced	N/A	N/A
Single Bond™ Universal Adhesive (3M)	Gilani et al., 2020 [33]	1%, 5%, 10% Ag-HA	Ag-HA nanoparticle powder	In Vitro	1% and 5%: significantly reduced; 10%: no significant difference	N/A	N/A

*: the bonding agent was tested in combination with AgNPs-modified composite for SBS.

### 3.3. Summary

It is important to emphasize that all currently existing studies on AgNP-incorporated bonding primers have been conducted in vitro and focused on targeting one single bacteria strain. This limitation restricts the evaluation of their effects on the complex biofilm inhabited on the tooth surface of patients. If and how saliva affects the release and function of AgNPs are unclear yet. Therefore, further ex vivo or in vivo studies are needed to confirm the efficacy of incorporating AgNPs into primers for reducing bacterial colonization while maintaining the bonding strength and natural teeth color in human usage [31]. However, the effects of AgNPs on SBS vary across studies, making it unclear whether these variations stem from differences in AgNP concentration, the physical and chemical interactions between AgNPs and various materials, or differing testing conditions. Furthermore, it remains uncertain whether the influence of AgNPs on SBS is consistent in an intraoral environment. These questions highlight the need for further investigation.

## 4. Nanosilver Particles in Orthodontic Composites

### 4.1. Antibacterial and Anti-Demineralization Effects of AgNPs

Multiple studies have suggested a diversity of benefits associated with incorporating specific concentrations of silver nanoparticles into orthodontic bonding composites (Table 3). As the clinically commonly used orthodontic bonding composite, Transbond™ XT (3M Unitek, Monrovia, CA, USA) is the most tested orthodontic composite with a broad range of concentrations of AgNPs [0.05–1% (*w*/*w*) in vitro and 1–10% (*w*/*w*) in vivo, respectively]. For instance, Eslamian et al. incorporated 0.3% (*w*/*w*) AgNPs into Transbond™ XT and found that its antibacterial effect against *S. mutans* did not significantly decline between 24 h and 30-day test time points [35]. Marco Sánchez-Tito et al. mixed 0.05%, 0.1%, 0.5%, and 1% (*w*/*w*) AgNPs with Transbond™ XT and observed that the 0.5% and 1% AgNPs modified Transbond™ XT could inhibit the growth of *S. mutans* and *Lactobacillus acidophilus* in vitro [36,37]. In addition, Transbond™ XT with 1% AgNPs significantly decreased the area and depth of white spot lesions on the tooth surfaces in an in vitro microbiological caries induction experiment [37]. The anti-demineralization effects of AgNPs have been further tested and compared with titanium dioxide (TiO_2_) nanoparticles in an in vitro circulating microbial model inoculated with *S. mutans* and *Lacticaseibacillus Casei* [38]. Notably, although 0.5% (*w*/*w*) AgNPs and 1% (*w*/*w*) TiO_2_ nanoparticles exhibited comparable anti-demineralization effects at a distance of 25–30 μm from the brackets, AgNPs demonstrated sustained anti-demineralization potential at a distance of 1.5 mm from the brackets, where the efficacy of TiO_2_ nanoparticles diminished [38]. Thus, based on the currently available in vitro studies, when incorporated with Transbond™ XT, a higher concentration (1%) of AgNPs is expected to provide long-lasting antibacterial and anti-demineralization effects, outperforming TiO_2_ nanoparticles. The antibacterial efficacy of AgNPs-modified Transbond™ XT was further assessed in vivo using Wistar rats [24]. In this study, all three tested concentrations [1%, 5%, and 10% (*w*/*w*)] of AgNPs markedly reduced the colony count of *S. mutans* in a dose-dependent manner. However, only 5% and 10% (*w*/*w*) AgNPs were effective in significantly lowering the colony count of *Streptococcus sanguinis* and *L. acidophilus* [24].

The antibacterial properties of AgNPs have also been evaluated in other orthodontic composites, such as Enlight Light Cure Composite (Ormco, Orange, CA, USA) [39] and Light-Cured experimental composite adhesive [40]. Again, AgNPs demonstrated superior antibacterial effects against *S. mutans* and *L. acidophilus* compared to TiO_2_ nanoparticles, as demonstrated by the disk agar diffusion test over a 30-day period [39]. Moreover, when compared to conventional Transbond™ XT and RMGI (Fuji Ortho LC, GC Corporation, Tokyo, Japan), experimental composite adhesives containing silica nanofillers and AgNPs displayed AgNP dose-dependent anti-adhesion effects against *S. mutans* and *Streptococcus sobrinus* [40].

The application of AgNPs was further explored in flowable composites. For instance, the addition of 1%, 2%, or 5% (*w*/*w*) AgNPs in Flow Tain composite resin (Reliance, Scottsdale, AZ, USA) significantly inhibited biofilm formations by *S. mutans*, *S. sanguinis*, and *L. acidophilus* in vitro, with the inhibitory effect exhibiting an AgNP dose-dependent pattern [8]. Similarly, incorporation of AgNPs at 0.5%, 1%, 2.5%, and 5% (*w*/*w*) into Master-Dent^®^ Flow Composite (Dentonics Inc.) also significantly reduced *S. mutans* colony counts, with the inhibition effect increasing proportionally with the AgNP concentration [29].

**Table 3:** The antibacterial properties of the silver nanoparticle (AgNPs)-added bonding composite. Conc.: concentration; *w*/*w*: weight per weight; WSL: white spot lesion; N/A: not applicable.

Composite	References	Tested Conc. of AgNPs (*w*/*w*)	Type of Study	Antibacterial Effect
Transbond^TM^ XT (3M)	Reddy et al., 2016 [41]	1%	In Vitro	N/A
Transbond^TM^ XT (3M)	Eslamian et al., 2020 [35]	0.3%	In Vitro	Long-lasting antibacterial effect on *S. mutans* at both 24 h and 30 days
Transbond^TM^ XT (3M)	Najafi et al., 2020 [38]	0.5%	In Vitro	Inhibit demineralization caused by *S. mutans* and *L. casei* up to 1.5 mm away from the brackets
Transbond^TM^ XT (3M)	Sánchez-Tito et al., 2021 [36]	0.05%, 0.1%, 0.5%, and 1%	In Vitro	Dose-dependently inhibit *S. mutans* and *L. acidophilus* and area of WSL
Transbond^TM^ XT (3M)	Sánchez-Tito et al., 2022 [42]	0.05%, 0.1%, 0.5%, and 1%	In Vitro	N/A
Transbond^TM^ XT (3M)	Sánchez-Tito et al., 2023 [37]	0.05%, 0.1%, 0.5%, and 1%	In Vitro	Does-dependently decrease the depth of the demineralization zone and area of WSL
Transbond^TM^ XT (3M))	Tavakolinejad et al., 2023 [43]	0.3%	In Vitro	N/A
Transbond^TM^ XT (3M)	Sánchez-Tito et al., 2024 [44]	0.05%, 0.1%, 0.5%, and 1%	In Vitro	N/A
Transbond^TM^ XT (3M)	Bahador et al., 2020 [24]	1%, 5%, 10%	In Vivo (Wistar Rats)	Dose-dependently inhibit *S. mutans*, *S. sanguinis*, and *L. acidophilus* at 24 h
Enlight Light Cure Composite (Ormco, Orange, CA, USA)	Mahendra et al., 2022 [39]	1%	In Vitro	Long-lasting antibacterial effect on *S. mutans* and *L. acidophilus* up to 30 days
Light-cured experimental composite adhesive	Ahn et al., 2009 [40]	0 ppm, 250 ppm, 500 ppm	In Vitro	Antibacterial effect by decreased *S. mutans* and *S. sobrinus* adhesion, and prevention of WSL
Flow Tain (Reliance Orthodontic Products, Inc., Itasca, IL, USA)	Mirhashemi et al., 2021 [8]	1%, 2%, 5%	In Vitro	Dose-dependently inhibit *S. mutans*, *S. sanguinis*, and *L. acidophilus*
Flow-It™ ALC™ Flowable Dental Composite (Pentron Clinical Technologies LLC, Orange, CA, USA)	Al-Thomali et al., 2022 [45]	0.05%	In Vitro	N/A
Master-Dent^®^ Flow Composite (Dentonics Inc.)	Jenabi et al., 2023 [29]	0.5%, 1%, 2.5%, and 5%	In Vitro	Dose-dependently inhibit *S. mutans*

### 4.2. Antibacterial Effects of Modified AgNPs

Several studies have also explored the antibacterial effectiveness of the combination of AgNPs with various particles (Table 4), and combining AgNPs with other antibacterial/anti-demineralization particles is the most common strategy. For example, an orthodontic adhesive containing nanoparticles of amorphous calcium phosphate-polydopamine-Ag (NPA) was developed to integrate the calcium phosphate remineralization system with the antibacterial Ag particles [46]. Adding NPA at concentrations as low as 0.2% (*wt*/*wt*) to Transbond™ XT demonstrated effective antimicrobial activities against *S. mutans* in vitro [46]. In another study, Aguiar et al. investigated a novel composite material by incorporating silicon dioxide-coated silver nanoparticles (Ag@SiO_2_-NPs) into Transbond™ XT and found that Ag@SiO_2_-NPs enhanced antibacterial properties against *S. mutans* [19]. Moreover, incorporating silver-doped zirconium dioxide nanoparticles (ZrO_2_AgDNPs) to Transbond™ XT also led to a significant reduction in *S. mutans* colony counts compared to control groups [47].

Since AgNPs are prone to self-agglomerate [48], which can significantly reduce their antibacterial efficacy, another widely adopted modification strategy focuses on controlling AgNP agglomeration and optimizing Ag^+^ release kinetics. For instance, graphene nanoplatelets (GNPs)-combined AgNPs were incorporated with Transbond™ XT, significantly inhibiting biofilm formation by *S. mutans* [49]. In another study, Kamran et al. loaded AgNPs into poly-l-glycolic acid (PLGA) nanoparticles, which exhibited a pH-sensitive, slow-releasing profile and significantly diminished the viability of *S. mutans* on Transbond™ XT in the short term (24 h) and long term (30 days) [50]. Additionally, to achieve a controlled release of silver ions over time, nano-bioactive glass-silver (nBG@Ag) was used to modify GC Ortho Connect adhesive (GC Orthodontics, Tokyo, Japan), which exhibited effective antibacterial potency against *S. mutans* in vitro [51].

**Table 4:** The antibacterial properties of the bonding composite with modified forms of silver nanoparticles (AgNPs). Conc.: concentration; *w*/*w*: weight per weight; WSL: white spot lesion; NPA: nanoparticles of amorphous calcium phosphate-polydopamine-Ag; Nacp: nanoparticles of amorphous calcium phosphate; GNP: graphene nanoparticles; NSPs-loaded PLGA: nanosilver particles loaded with poly-L-glycolic acid; PVA: polyvinyl alcohol; TCM: chloroform; Ag-HA NPs: silver hydroxyapatite nanoparticles; *v*/*v*%: Volume/volume percentage; Ag@SiO_2_NPs: silicon dioxide-coated silver nanoparticles; ZrO_2_AgDNPs: Zirconium dioxide silver-doped nanoparticles; β-AgVO_3_: Nanostructured silver vanadate decorated with silver nanoparticles; nBG@Ag: nano-bioactive glass-silver.

Bonding Reagent	References	Tested Conc. of AgNPs (*w*/*w*)	Combinatory Materials	Type of Study	Antibacterial Effect
Transbond^TM^ XT	Jia et al., 2023 [46]	NPA: 0.1%, 0.2%, 0.3%, and 0.5%	50 mM AgNO_3_ mixed with 50 mg Nacp + 2 mg/mL dopamine hydrochloride	In Vitro	Inhibit *S. mutans* growth, prevent WSL (Only tested with 0.2%)
Transbond^TM^ XT	Sawan et al., 2021 [49]	GNP-Ag: 0.25%, 0.5%	80 mg AgNO_3_ mixed with 50 mg GNP solution	In Vitro	Dose-dependently inhibit *S. mutans* at 24 h and 30 days
Transbond^TM^ XT	Kamran et al., 2022 [50]	NSPs-loaded PLGA: 2.5%, 5%	0.2 mL of 10 mM AgNO_3_ mixed with 2 mg of 0.5% PVA + 1 mL of NaBH_4_ + 10 mg of PLGA in 1.5 mL of TCM	In Vitro	Dose-dependently inhibit *S. mutans* at 24 h and 30 days
Transbond^TM^ XT	Sodagar et al., 2016 [52]	Ag-HA NPs: 1%, 5%, and 10%	100 mg AgNO_3_ mixed with 1 g HA nano powder	In Vitro	Dose-dependent (5% and 10% are similar) antibacterial effect on *S. mutans*, *L. acidophilus*, and *S. sanguinis* at 3, 15, 30 days; prevent WSL
Transbond^TM^ XT	Rajan et al., 2024 [53]	Ag-HA NPs: 2%, 4% (*v*/*v*%)	1 g of nanosized HA powder + Ag in 100 mL of ethanol + NH_4_H_2_PO_4_ + Ammonium hydroxide + AgNO_3__Ca(NO_3_)24H_2_O	In Vitro	Dose-dependent antibacterial effect on *S. aureus*, *S. mutans*, and *E. coli*
Transbond^TM^ XT	Aguiar et al., 2022 [19]	Ag@SiO_2_NPs: 0.5%, 1%, 3%	Ag@SiO_2_NPs	In Vitro	Dose-dependent antibacterial effect on *S. mutans*
Transbond^TM^ XT	Almoammar et al., 2024 [47]	ZrO_2_AgDNP: 2.5%, and 5%	ZrO_2_AgDNP	In Vitro	Dose-dependent antibacterial effect on *S. mutans*
Transbond^TM^ XT	Uehara et al., 2024 [54]	β-AgVO_3_: 2.5%, 5%	β-AgVO_3_	In Vitro	Dose-dependent antibacterial effect on *S. mutans* and *S. sanguinis*
No-mix self-cure composite resin (Unite Bonding System; Reliance, USA)	Kachoei et al., 2021 [55]	Ag/ZnO: 5%, 10%, 15%, and 20%	AZ: Ag + ZnO synthesized; AZ: ZnO nanoparticle + AgNO_3_ solution	In Vitro	All Conc. showed antibacterial activity against *S. mutans*, *S. aureus*, *E. coli*, and *L. gasseri*; All Conc. has no effect against *Candida albicans*
GC Ortho Connect (GC Orthodontics, Japan)	Seifi et al., 2024 [51]	nBG@Ag: 1%, 3%, 5%	2000M 2% PEG + di-ammonium hydrogen orthophosphate + AgNO_3_	In Vitro	Dose-dependent antibacterial effect on *S. mutans*

Moreover, several studies have assessed the antibacterial effects of modified AgNP systems against the polymicrobial species involved in white spot lesion formation. For instance, Ag/HA nanoparticles in Transbond™ XT composite significantly reduced the growth of *S. mutans*, *L. acidophilus*, *S. sanguinis, Staphylococcus aureus*, and *Escherichia coli*, thereby exhibiting high efficiency against white spot formation [52,53]. Meanwhile, modifying Transbond™ XT with nanostructured silver vanadate decorated with silver nanoparticles (βAgVO_3_) resulted in a marked reduction in *S. mutans* and *S. aureus* colony-forming units (CFUs) [54]. Since AgNPs can increase the surface roughness of orthodontic adhesives, a key factor that promotes bacterial adhesion [56], nanofillers have been recruited in the experimental composite adhesives to mitigate this issue [40]. Ahn et al. found that orthodontic adhesives containing AgNP-impregnated nanofillers have potent inhibitory effects on the adhesion and proliferation of cariogenic streptococci *S. mutans* and *S. sobrinus* in both non-saliva-coating and saliva-coating environments [40]. However, these AgNP-impregnated nanofiller composites failed to form the inhibition zone in the disk diffusion test [40], indicating a relatively low antibacterial effect compared to other types of AgNP composites. In a study aimed at developing a novel bioactive composite resin, Ag and zinc oxide (ZnO) were combined and integrated into a no-mix self-cure composite resin (Unite Bonding System; Reliance, USA) to synergize the antibacterial effects while minimizing the aesthetic concerns of Ag since ZnO imparts a white color [55]. Among the composites [control (no ZnO or Ag) group, ZnO nanoparticle-only group, AZ group with ZnO nanoparticles and silver ions, and AZ group with Ag/ZnO nanoparticles], the AZ group demonstrated the highest antimicrobial activity against *S. mutans*, *S. aureus*, *Lactobacillus gasseri*, and *E. coli*, but not *Candida albicans* [55].

### 4.3. Side Effects

#### 4.3.1. Influences on Shear Bond Strength (SBS)

Numerous studies have consistently demonstrated a dose-dependent incorporation of AgNPs, either alone (Table 5) or in modified forms (Table 6), to decrease the SBS of Transbond™ XT [19,35,41,43,44,46,49,50,54], except ZrO_2_AgDNP, which increases the micro SBS of Transbond™ XT with dose-dependency [47]. Reddy et al. noted that, compared to ZnO or TiO_2_ nanoparticles, AgNPs have less influence on the SBS of Transbond™ XT [41]. On the contrary, Mahendra et al. observed a greater reduction in the SBS of Enlight composite (Ormco Corp, Brea, CA, USA) resulting from AgNP incorporation than that from TiO_2_ incorporation [39]. Furthermore, nano-bioactive glass-silver (nBG@Ag) reduced the SBS of GC Ortho Connect (GC Orthodontics, Japan) [51]. However, AgNPs did not considerably alter the SBS of light-cured experimental composite adhesives [40], nor did Ag/ZnO nanoparticles on no-mix self-cure composite resin (Unite Bonding System; Reliance, USA) [55].

The varying effects of AgNPs on SBS also extended to flowable composites. Yousef Al-Thomali reported that AgNPs significantly increased the SBS of the Nano-Bond Flow-It™ ALC™ Flowable Dental Composite (Pentron Clinical Technologies LLC., Orange, CA, USA) under different cyclic loading and thermal loading consistently [45]. On the other hand, Jenabi et al. [29] found that the SBS of fiber-reinforced composite (Master-Dent^®^ Flow Composite (Dentonics Inc.) decreased only when the concentration of the incorporated AgNPs reached 5% (*w*/*w*), with no significant reduction observed at lower concentrations [29].

#### 4.3.2. Discoloration

Research on enamel discoloration caused by incorporating AgNPs into orthodontic composites remains limited to date. However, a recent study by Sanchez-Tito et al. found that higher concentrations of the incorporated AgNPs were associated with a greater darkening of the teeth [42] (Table 5 and Table 6).

#### 4.3.3. Cytotoxicity

While AgNPs alone exhibit promising biocompatibility (Table 5), recent studies have investigated the cytotoxicity of modified AgNPs to evaluate the potential risks introduced by additional components in the composition (Table 6). For example, Jia et al. reported that NPA fillers supported the growth of L929 cells, a fibroblast cell line, with 70% cell viability in vitro [46]. Similarly, Transbond™ XT modified with 0.25% (*w*/*w*) GNP-Ag exhibited over 80% cell viability when tested with human gingival fibroblasts (HGFs); however, cell survival rates decreased as the GNP-Ag concentration increased [49]. Kamran et al. [50] reported that 2.5% (*w*/*w*) NSPs-loaded PLGA nanoparticles promoted the proliferation of HGF cells, while when the concentration increased to 5% (*w*/*w*), the viability of HGF cells decreased [50]. Other studies have shown that Ag/ZnO nanoparticles exhibited no adverse effects on HGF viability at concentrations up to 0.1 µg/mL [55]. Additionally, nBG@Ag incorporated into GC Ortho Connect (GC Orthodontics, Japan) adhesive composites at concentrations up to 5% (*w*/*w*) showed no cytotoxicity to cellular structures [51]. Overall, these modified AgNPs generally exhibit low cytotoxicity, thereby enhancing their potential for safe use in orthodontic practice.

**Table 5:** The side effects of the silver nanoparticle (AgNP)-added bonding composite. Conc.: concentration; *w*/*w*: weight per weight; SBS: shear bonding strength; N/A: not applicable.

Composite	References	Tested Conc. of AgNPs (*w*/*w*)	Type of Study	Side Effects		
				SBS	Cytotoxicity	Discoloration
Transbond^TM^ XT (3M)	Reddy et al., 2016 [41]	1%	In Vitro	Decreased	N/A	N/A
Transbond^TM^ XT (3M)	Eslamian et al., 2020 [35]	0.3%	In Vitro	Decreased	N/A	N/A
Transbond^TM^ XT (3M)	Najafi et al., 2020 [38]	0.5%	In Vitro	N/A	N/A	N/A
Transbond^TM^ XT (3M)	Sánchez-Tito et al., 2021 [36]	0.05%, 0.1%, 0.5%, and 1%	In Vitro	N/A	N/A	N/A
Transbond^TM^ XT (3M)	Sánchez-Tito et al., 2022 [42]	0.05%, 0.1%, 0.5%, and 1%	In Vitro	N/A	N/A	Dose-dependent enamel discoloration after 6 months
Transbond^TM^ XT (3M)	Sánchez-Tito et al., 2023 [37]	0.05%, 0.1%, 0.5%, and 1%	In Vitro	N/A	N/A	N/A
Transbond^TM^ XT (3M))	Tavakolinejad et al., 2023 [43]	0.3%	In Vitro	Decreased	N/A	N/A
Transbond^TM^ XT (3M)	Sánchez-Tito et al., 2024 [44]	0.05%, 0.1%, 0.5%, and 1%	In Vitro	Decreased	N/A	N/A
Transbond^TM^ XT (3M)	Bahador et al., 2020 [24]	1%, 5%, 10%	In Vivo (Wistar Rats)	N/A	N/A	N/A
Enlight Light Cure Composite (Ormco)	Mahendra et al., 2022 [39]	1%	In Vitro	Decreased	N/A	N/A
Light-cured experimental composite adhesive	Ahn et al., 2009 [40]	0 ppm, 250 ppm, 500 ppm	In Vitro	No significant difference	N/A	N/A
Flow Tain (Reliance Orthodontic Products, Inc.)	Mirhashemi et al., 2021 [8]	1%, 2%, 5%	In Vitro	N/A	N/A	N/A
Flow-It™ ALC™ Flowable Dental Composite (Pentron Clinical Technologies LLC.)	Al-Thomali et al., 2022 [45]	0.05%	In Vitro	Increased	N/A	N/A
Master-Dent^®^ Flow Composite (Dentonics Inc.)	Jenabi et al., 2023 [29]	0.5%, 1%, 2.5%, and 5%	In Vitro	Only significant reduction in 5%	N/A	N/A

**Table 6:** The side effects of the bonding composite with modified forms of silver nanoparticles (AgNPs). Conc.: concentration; *w*/*w*: weight per weight; SBS: shear bonding strength; NPA: nanoparticles of amorphous calcium phosphate-polydopamine-Ag; Nacp: nanoparticles of amorphous calcium phosphate; GNPs: graphene nanoparticles; HGF: human gingival fibroblast; NSPs-loaded PLGA: nanosilver particles loaded with poly-L-glycolic acid; PVA: polyvinyl alcohol; TCM: chloroform; Ag-HA NPs: silver hydroxyapatite nanoparticles; *v*/*v*%: Volume/volume percentage; Ag@SiO_2_NPs: silicon dioxide-coated silver nanoparticles; ZrO_2_AgDNPs: Zirconium dioxide silver-doped nanoparticles; µTBS: micro tensile bond strength; β-AgVO_3_: Nanostructured silver vanadate decorated with silver nanoparticles; nBG@Ag: nano-bioactive glass-silver; N/A: not applicable.

Bonding Reagent	References	Tested Conc. of AgNPs (*w*/*w*)	Combinatory Materials	Type of Study	Side Effects			
					SBS	Cytotoxicity	Discoloration	Surface Roughness
Transbond^TM^ XT	Jia et al., 2023 [46]	NPA: 0.1%, 0.2%, 0.3%, & 0.5%	50 mM AgNO_3_ mixed with 50 mg Nacp + 2 mg/mL dopamine hydrochloride	In Vitro	0.1% and 0.2%: met the minimal standard SBS; 0.3% and 0.5%: significantly reduced	All conc. showed greater than 70% cell viability (L929 cells)	N/A	N/A
Transbond^TM^ XT	Sawan et al., 2021 [49]	GNP-Ag: 0.25%, 0.5%	80 mg AgNO_3_ mixed with 50 mg GNP solution	In Vitro	0.25%: no significant effect; 0.5%: decreased	0.25%: >80% HGF survival; 0.5%: <80% HGF survival after 48 h;	N/A	N/A
Transbond^TM^ XT	Kamran et al., 2022 [50]	NSPs-loaded PLGA: 2.5%, 5%	0.2 mL of 10 mM AgNO_3_ mixed with 2 mg of 0.5% PVA + 1 mL of NaBH_4_ + 10 mg of PLGA in 1.5 mL of TCM	In Vitro	2.5%: no significant effect; 5%: decreased	2.5%: increased HGF viability rate after 24, 48, and 72 h; 5%: decreased HGF viability in 48 h and 72 h	N/A	N/A
Transbond^TM^ XT	Sodagar et al., 2016 [52]	Ag-HA NPs: 1%, 5%, and 10%	100 mg AgNO_3_ mixed with 1 g HA nano powder	In Vitro	N/A	N/A	N/A	N/A
Transbond^TM^ XT	Rajan et al., 2024 [53]	Ag-HA NPs: 2%, 4% (*v*/*v*%)	1 g of nanosized HA powder + Ag in 100 mL of ethanol + NH_4_H_2_PO_4_ + Ammonium hydroxide + AgNO_3__Ca(NO_3_)24H_2_O	In Vitro	N/A	N/A	N/A	N/A
Transbond^TM^ XT	Aguiar et al., 2022 [19]	Ag@SiO_2_NPs: 0.5%, 1%, 3%	Ag@SiO_2_NPs	In Vitro	No significant difference to the control; but 3% had significant lower SBS than 1%	N/A	N/A	N/A
Transbond^TM^ XT	Almoammar et al., 2024 [47]	ZrO_2_AgDNP: 2.5%, & 5%	ZrO_2_AgDNP	In Vitro	Dose-dependent increase in µTBS	N/A	N/A	N/A
Transbond^TM^ XT	Uehara et al., 2024 [54]	β-AgVO_3_: 2.5%, 5%	β-AgVO_3_	In Vitro	Decreased in both concentrations regardless of thermocycling	N/A	N/A	Dose-dependently decreased
No-mix self-cure composite resin (Unite Bonding System; Reliance, USA)	Kachoei et al., 2021 [55]	Ag/ZnO: 5%, 10%, 15%, and 20%	AZ: Ag + ZnO synthesized; AZ: ZnO nanoparticle + AgNO_3_ solution	In Vitro	No significant difference	No effect on HGF viability up to 0.1 µg/mL for AZ group, AZ group had lowest viability	N/A	N/A
GC Ortho Connect (GC Orthodontics, Japan)	Seifi et al., 2024 [51]	nBG@Ag: 1%, 3%, 5%	2000 M 2% PEG + di-ammonium hydrogen orthophosphate + AgNO_3_	In Vitro	Decreased within clinically acceptable range	No significant effect	N/A	N/A

### 4.4. Summary

Numerous studies have conclusively demonstrated that incorporating AgNPs, either alone or in modified forms, into the composite resins yields significant short-term antibacterial effectiveness in a dose-dependent manner. However, it is noteworthy that many current studies have been limited to single-species bacterial tests, predominantly focusing on *S. mutans*. In addition, various intraoral factors, such as thermal fluctuation, saliva composition, saliva flow, and acid–base balance, as well as other individual patient characteristics, which were not accounted for in most in vitro studies, can influence the performance of AgNPs [8]. Therefore, further investigations, particularly well-designed animal studies and clinical trials, are warranted to evaluate the in vivo effects of AgNPs on oral microbiota and to determine whether the antimicrobial effects of AgNPs can be sustained in the intraoral environment over time. In addition, agglomeration poses a significant challenge that can undermine the efficacy of AgNPs [48]. Targeted research on stabilization techniques, such as polymer coatings and encapsulation methods, is crucial to improve the dispersion and controlled release of AgNPs. Moreover, there is a notable research gap concerning enamel discoloration associated with modified AgNPs. Comprehensive studies are needed to evaluate the extent and impact of discoloration caused by these nanoparticles. By addressing these critical challenges, modified AgNPs are poised to become integral components of next-generation orthodontic composites, providing sustained antibacterial activities while preserving optimal mechanical and aesthetic properties.

## 5. Nanosilver Particles in Glass Ionomer Cement

### 5.1. Antibacterial Effects

AgNPs have also been incorporated into glass ionomer cement (GIC) and resin-modified glass ionomer cement (RMGIC) to arrest caries and prevent bacterial biofilm formation on the surface of bonding reagents (Table 7). For instance, Paiva et al. reported that AgNPs-incorporated GIC inhibited the growth of *E. coli* and reduced the metabolic activity of *S.mutans* biofilms [57]. Notably, these effects were dose-dependent, with 0.50% (*w*/*w*) AgNPs achieving a remarkable 99% reduction in bacterial viability compared to the negative control group. Similarly, Wang et al. found that incorporating AgNPs into RMGIC lowered the metabolic activity and lactic acid production of *S. mutans*, decreased the total counts of streptococci and planktonic bacteria, and prohibited biofilm formation [58]. The antibacterial activities of the AgNPs-incorporated RMGIC also exhibit a dose-dependent pattern and can reduce white spot lesion formation around brackets in vitro [58].

Meanwhile, other combinatory materials were also used with AgNPs for RMGIC modification. For example, a nanosilver base inorganic antibacterial powder (AgNaZr_2_(PO_4_)3·H2O; AGP-ZP003) exhibited a dose-dependent antibacterial effect against *S.mutans* both in vitro [59] and in vivo [60]. However, long-term storage may reduce the antibacterial effectiveness of AGP-ZP003-modified RMGIC, requiring further investigation on how to improve the stability of this material [59]. Notably, Ding et al. incorporated N-acetylcysteine (NAC) alongside AgNPs into RMGIC, resulting in lower CFU counts of *S.mutans* compared to formulations that did not include NAC [61].

**Table 7:** The antibacterial properties of the glass ionomer cement with silver nanoparticles (AgNPs). Conc.: concentration; *w*/*w*: weight per weight; WSL: white spot lesion; GIC: glass ionomer cement; RMGIC: resin-modified glass ionomer cement; NAC: N-acetylcysteine; Si-HA-Ag: Silica-hydroxyapatite-silver; AgNaZr_2_(PO_4_)_3_·H_2_O, AGP-ZP003: nanosilver base inorganic antibacterial powder; N/A: not applicable.

Bonding Reagent	References	Tested Conc. of AgNPs (*w*/*w*)	Combinatory Materials	Type of Study	Antibacterial Effect
GIC (Queen Mary University of London)	Paiva et al., 2018 [57]	0.05%, 0.10%, and 0.50%	N/A	In Vitro	dose-dependent antibacterial effect against *S. mutans* and *E. coli*
GIC (GC Fuji II)	Jowkar et al., 2019 [62]	0.1%, 0.2%	N/A	In Vitro	N/A
RMGIC (GC LC Fuji)	Wang et al., 2015 [58]	0.05%, 0.1%	N/A	In Vitro	dose-dependent effects on against *S. mutans*, total streptococci, and planktonic bacteria; and on reduced WSL
RMGIC (GC LC Fuji)	Ding et al., 2021 [61]	0.15%	AgNPs + 0%, 5%, 10%, 20%, 30% NAC	In Vitro	AgNPs alone group inhibits *S. mutans*; 20% NAC increased the AgNPs’ effects against *S. mutans*
RMGIC (GC LC Fuji II)	Raghimi et al., 2024 [63]	0.1%, 0.5%, 1% and 2%	Si-HA-Ag hybrid nanoparticles	In Vitro	N/A
RMGIC (GC LC Fuji)	Biglar et al., 2023 [64]	2%, 5%, 10%	Si-HA-Ag hybrid nanoparticles	In Vitro	N/A
RMGIC (GC LC Fuji)	Li et al., 2013 [59]	1%, 3%, 5%, 10%, 15%	AgNaZr_2_(PO_4_)_3_·H_2_O, AGP-ZP003	In Vitro	Dose-dependent antibacterial effect against *S. mutans*
RMGIC (GC LC Fuji)	Li et al., 2015 [60]	1%, 3%, 5%, 10%, 15%	AgNaZr_2_(PO_4_)_3_·H_2_O, AGP-ZP003	In Vivo (SD rats)	Dose-dependent bactericidal effect against *S. mutans*

### 5.2. Side Effects

#### 5.2.1. SBS

The impact of AgNPs on SBS values appears to vary among different types of cements (Table 8), as previous studies have indicated that incorporating AgNPs at concentrations of 0.1% and 0.2% (*w*/*w*) increased the SBS of GIC with dentin [62], where the same concentrations did not affect the SBS of RMGIC [58]. However, in the study conducted by Ding et al., when 30% NAC was incorporated alongside AgNPs at a concentration of 0.15% (*w*/*w*), the SBS of RMGIC was significantly reduced, although lower concentrations of NAC did not result in any noticeable changes [61]. Furthermore, a dose-dependent decrease in SBS was observed when AGP-ZP003 was incorporated into GMGIC [59]. Similarly, silica-hydroxyapatite-silver (Si-HA-Ag) hybrid nanoparticles, which were explored due to HA’s potential ability to enhance the biocompatibility and mechanical properties of glass ionomer materials, also showed a statistically significant decrease in SBS at the concentration of 10% (*w*/*w*) but not at concentrations of 2% and 5% (*w*/*w*) [64].

#### 5.2.2. Cytotoxicity

Currently, the evaluation of cytotoxicity for AgNP-modified glass ionomers is also limited (Table 8). However, Ding et al. revealed that incorporating 0.15% (*w*/*w*) AgNPs into RMGIC significantly reduced the viability of HGFs in vitro. Notably, this inhibitory effect was partially rescued by co-incorporating 20% NAC, indicating that using combinatory materials has the potential to enhance the biocompatibility of AgNP-modified glass ionomer materials [61].

#### 5.2.3. Discoloration

Research on the discoloration effects of AgNPs on glass ionomer materials is also scarce and can hardly be compared head-to-toe (Table 8). Wang et al. concluded that incorporating 0.05% and 0.1% (*w*/*w*) AgNPs into RMGIC caused no noticeable color change [58]. On the contrary, incorporating Si-HA-Ag hybrid nanoparticles or AGP-ZP003 into RMGIC led to observable discoloration, with varying degrees of intensity reported [59,63].

**Table 8:** The side effects of the glass ionomer cement with silver nanoparticles (AgNPs). Conc.: concentration; *w*/*w*: weight per weight; SBS: shear bonding strength; GIC: glass ionomer cement; RMGIC: resin-modified glass ionomer cement; NAC: N-acetylcysteine; Si-HA-Ag: Silica-hydroxyapatite-silver; AgNaZr_2_(PO_4_)_3_·H_2_O, AGP-ZP003: nanosilver base inorganic antibacterial powder; N/A: not applicable.

Bonding Reagent	References	Tested Conc. of AgNPs (*w*/*w*)	Combinatory Materials	Type of Study	Side Effects		
					SBS	Cytotoxicity	Discoloration
GIC (Queen Mary University of London)	Paiva et al., 2018 [57]	0.05%, 0.10%, and 0.50%	N/A	In Vitro	N/A	N/A	N/A
GIC (GC Fuji II)	Jowkar et al., 2019 [62]	0.1%, 0.2%	N/A	In Vitro	dose-dependent increase (to dentin)	N/A	N/A
RMGIC (GC LC Fuji)	Wang et al., 2015 [58]	0.05%, 0.1%	N/A	In Vitro	No significant effect	N/A	No noticeable change
RMGIC (GC LC Fuji)	Ding et al., 2021 [61]	0.15%	AgNPs + 0%, 5%, 10%, 20%, 30% NAC	In Vitro	0–20% NAC: no effect; 30% NAC: decreased	AgNPs: reduced cell viability; AgNPs + 20%NAC: increased cell viability than AgNPs alone	N/A
RMGIC (GC LC Fuji II)	Raghimi et al., 2024 [63]	0.1%, 0.5%, 1% and 2%	Si-HA-Ag hybrid nanoparticles	In Vitro	N/A	N/A	Dose-dependent increased yellowish-brown
RMGIC (GC LC Fuji)	Biglar et al., 2023 [64]	2%, 5%, 10%	Si-HA-Ag hybrid nanoparticles	In Vitro	2%: Slightly increased, no significant effect; 5%: Slightly decreased, no significant effect; 10%: Significantly decreased	N/A	N/A
RMGIC (GC LC Fuji)	Li et al., 2013 [59]	1%, 3%, 5%, 10%, 15%	AgNaZr_2_(PO_4_)_3_·H_2_O, AGP-ZP003	In Vitro	Dose-dependent decrease and was significant in 15% nanosilver but within the clinical acceptable range	N/A	very light grey color for all conc.
RMGIC (GC LC Fuji)	Li et al., 2015 [60]	1%, 3%, 5%, 10%, 15%	AgNaZr_2_(PO_4_)_3_·H_2_O, AGP-ZP003	In Vivo (SD rats)	N/A	N/A	N/A

### 5.3. Summary

Overall, incorporating AgNPs into GICs for bonding applications shows considerable promise, particularly regarding antibacterial properties. The combination with other materials, such as NAC and silica-hydroxyapatite, further improves their efficacy and biocompatibility. However, several challenges persist in their clinical application, including concerns related to SBS, cytotoxicity, and discoloration. It is also worth noting that the mixing process of GIC often introduces air inclusion, which can compromise its mechanical integrity. Incorporating small-sized AgNPs into GIC could address this issue by filling the spaces between larger glass particles, improving the packing process, reducing air entrapment, and providing additional bonding sites for the polyacrylic polymer. This approach may enhance the physical properties of GIC, such as flexural strength, compressive strength, and surface microhardness [62]. Furthermore, future research should evaluate the chemical interactions between AgNPs and GIC components, the potential effects of nanoparticle incorporation on the setting time of GIC, and the patterns of fluoride ion release [62].

## 6. Future Directions

### 6.1. Limitations of Currently Available Investigations

Numerous studies have investigated the antibacterial effects of AgNPs, either alone or in modified forms, incorporated into orthodontic bonding reagents. However, most of these studies are limited to in vitro experiments and target single bacteria species. The influence of intraoral environments and the presence of metal orthodontic appliances on the property of the AgNPs-modified bonding reagents remained largely unknown. In addition, although AgNPs show promising antibacterial effects over a 30-day test period, the clinical significance of this duration is uncertain, given that orthodontic treatment typically lasts years and rebonding patients every 30 days is impractical. Nevertheless, with the broad use of clear aligners, it has been shown that beta diversities of oral microbial communities differ between patients with fixed appliances and those with clear aligners [65]. Thus, whether AgNPs-modified bonding reagents can hold their antibacterial effects on clear aligner-associated predominant pathogenic bacteria when used on limited tooth surfaces for attachments is an interesting question to be answered. It is also critical to assess whether AgNPs can cause damage to the aligners in ways that may compromise orthodontic treatment outcomes.

### 6.2. Future Directions

Despite these uncertainties, AgNPs hold strong potential for helping to prevent caries and WSL during orthodontic treatment. A worldwide collaboration is warranted to advance the development of AgNPs-based materials in orthodontics by expanding in vivo tests, as well as by understanding the interaction between AgNPs released from the bonding reagents and orthodontic appliances, especially in the intraoral environment.

## Data Availability

No new data were created or analyzed in this study. Data sharing is not applicable to this article.
